# Pellagra

**Published:** 1909-09

**Authors:** 


PELLAGRA.—Pellagra, a disease occurring in Italy, Southern France and Spain, and attributed to the use of diseased maize, hitherto unknown in this country, has recently gained a foothold in the Southern States and is reported to be on the increase.

It is characterized in the early stages by debility, spinal pains and digestive disturbances; later erythema develops, with drying and exfoliation of the skin. In severe cases various mental manifestations arise, such as spasms, ataxic paraplegia and mental disturbances. In cases presenting ataxic paraplegia the spinal cord has shown combined posterior and lateral sclerosis. The patient at first feels unfit for work, suffers from headache, giddiness and a burning of the skin, associated occasionally with a red or livid rash, which is very painful. The patient's condition improves during the winter months, but as soon as spring reappears the symptoms become exaggerated, the nervous system suffers a breakdown, and as the disease progresses the patient passes into a state of hopeless insanity or imbecility. The disease is more prevalent in the spring, decreasing as fall comes on.

A commission from the United States Department of Agriculture is at present in Italy for the purpose of discovering the cause and means of preventing the disease.

Apropos the presence of pellagra in the South and with our close relationship, commercially and socially, with that section, it may not be inadvertent to note that Thayer (*Johns Hopkins Bulletin,* July, 1909) reports the occurrence of two cases in Maryland.
—*Maryland Medical Journal.*

